# Effects of a School-Based Physical Activity Intervention for Obesity and Health-Related Physical Fitness in Adolescents With Intellectual Disability: Protocol for a Randomized Controlled Trial

**DOI:** 10.2196/25838

**Published:** 2021-03-22

**Authors:** Aiwei Wang, Yang Gao, Jingjing Wang, Tomas K Tong, Yan Sun, Siyue Yu, Hong Zhao, Daozhi Zou, Ziheng Zhang, Yuling Qi, Nan Zuo, Danran Bu, Dexing Zhang, Yaojie Xie, Julien S Baker

**Affiliations:** 1 Department of Sport, Physical Education and Health Hong Kong Baptist University Hong Kong China; 2 Centre for Health and Exercise Science Research Hong Kong Baptist University Hong Kong China; 3 Mass Sports Research Center Institute of Sport Science Beijing China; 4 JC School of Public Health and Primary Care The Chinese University of Hong Kong Hong Kong China; 5 Qianjiang Special Education School Qianjiang China; 6 Zhijiang Special Education School Zhijiang China; 7 Dangyang Special Education School Dangyang China; 8 Yingshan Special Education School Yingshan China; 9 HuBei Institute of Sport Science Wuhan China; 10 School of Nursing Faculty of Health and Social Science The Hong Kong Polytechnic University Hong Kong China

**Keywords:** children, intellectual disability, physical activity, overweight, obesity, intervention

## Abstract

**Background:**

Childhood obesity accompanied by lower levels of health-related physical fitness (HRPF) is a major threat to public health both internationally and locally. Children with intellectual disability, especially adolescents, have a higher risk of being overweight/obese and having poor HRPF levels. Therefore, more interventions are needed to help this population attain their optimal health levels. However, there has been relatively limited research on this population compared with on their typically developing peers.

**Objective:**

The proposed study aims to fill this knowledge gap by developing and examining the success of a physical activity (PA) intervention for the target population.

**Methods:**

The proposed study will be a 12-week, school-based randomized controlled trial. The participants (N=48) will be recruited from special schools for students with mild intellectual disability and then randomly allocated to either the intervention group (IG) or the wait-list control group (CG). During the intervention period, the participants in the IG will receive a fun game–based moderate-to-vigorous PA (MVPA) training program (2 sessions/week, 60 minutes/session, for a total of 24 sessions). The intensity of the activities will increase in a progressive manner. Participants in the CG will receive no program during the study period, but the same PA program will be provided to them after the completion of the study. To observe and evaluate the sustaining effects of the intervention, follow-up testing will be scheduled for the participants 12 weeks after the intervention concludes. The study outcomes will include primary outcomes (obesity- and fitness-related outcomes) and a secondary outcome (blood pressure). All of the measurements will be taken at 3 time points. After the follow-up tests, the same PA training program will be provided to the participants in the CG.

**Results:**

This study is ongoing. The participants were recruited from October 2020 to November 2020. The total duration of the study is 13 months. Study results are expected at the end of 2021.

**Conclusions:**

The proposed study is expected to reduce obesity and improve HRPF levels in children with intellectual disability. If proven effective, the intervention will be made accessible to more special schools and mainstream schools with students with intellectual disability. Furthermore, the study can serve as an example for international researchers, policy makers, and members of the public who are seeking to tackle the problem of obesity and poor HRPF among children with intellectual disability.

**Trial Registration:**

ClinicalTrials.gov NCT04554355; https://www.clinicaltrials.gov/ct2/show/NCT04554355

**International Registered Report Identifier (IRRID):**

PRR1-10.2196/25838

## Introduction

Childhood obesity accompanied with lower levels of health-related physical fitness (HRPF) is a major threat to public health internationally and locally [1]. The global prevalence of overweight and obesity among children has risen dramatically from 4% in 1975 to over 18% in 2016 [2]. This rising trend has been recorded in a wide range of countries [3]. For example, in the United States, obesity prevalence increased from 13.5% in 1995 to 18.5% in 2016 [2]. In China, the prevalence of overweight and obesity increased from 1.1% in 1985 to 20.4% in 2014 [4]. In terms of HRPF levels, the most update-to-date data showed that only 42% to 46% of children had adequate cardiopulmonary fitness (CPF) [5]. Studies have also demonstrated a global decline in CPF levels of 0.36% per year [6]. There is clear evidence that childhood obesity accompanied with lower levels of HRPF leads to short- and long-term risks, including cardiovascular diseases, metabolic syndrome, osteoporosis, type 2 diabetes, hypertension, and certain types of cancer [7,8].

Children with intellectual disability, who account for approximately 1% of the entire pediatric population, are more vulnerable than their typically developing (TD) peers to obesity and lower levels of HRPF [9,10], as they tend to be less active and less empowered to choose and adopt healthy behaviors [3]. The global prevalence of overweight and obesity in children with intellectual disability is 30% to 33% [9], and the risk of developing overweight and obesity is 1.54 to 1.80 times higher in adolescent children with intellectual disability (aged 12 to 18 years) than in their TD peers [3]. In terms of HRPF levels, a cross-sectional study found that 71% to 91% of children with intellectual disability (aged 12 to 18 years) scored below the reference values (for TD children) in CPF and muscular fitness [11].

Therefore, given the high prevalence of obesity and poor HRPF levels, children with intellectual disability, especially adolescents, should be a target population for reducing obesity and improving HRPF levels. Compared with the large body of research on effective interventions for obesity and lower levels of HRPF among TD children, few efforts have been made to examine the effectiveness of interventions for children with intellectual disability. To date, 3 reviews have examined intervention effects on changes in body weight and/or HRPF levels [12-14] in this population. Harris et al [12] examined the effects of 6 physical activity (PA) interventions that were described as including aerobic and resistance activities. However, no significant effects were found on any obesity-related outcomes. The second review, by Maïano et al [13], focused on lifestyle interventions, including PA, diet, health education, and cognitive behavioral strategies. The review of 9 studies concluded that interventions involving PA were effective in reducing body weight, BMI, fat mass, and peak oxygen consumption per unit time/peak power, and increasing peak power, maximal upper and lower limb strength, and number of sit-ups. However, inconsistent results were found for body fat percentage, waist circumference, and peak heart rate [13]. A third review [14] examined the effects of 6 interventions for excessive weight among children with intellectual disability. The intervention approaches included PA and parental engagement. However, no consistent results were found for any of the intervention types.

Based on the 3 reviews, we cannot draw any firm conclusions on intervention effects because of the limited number of studies involved. In addition, the most recent of the 3 reviews was published in 2014. Since 2014, there has been an increase in the number of interventions in the target population. Therefore, an updated review on the topic is needed to identify effective interventions for this population.

Hence, given the above evidence, we conducted a systematic review and meta-analysis on this topic (data have not yet been published). Our findings suggested the following: (1) PA was the predominant component to be adopted into interventions, with 27 of the 29 identified interventions implementing PA; (2) PA contributes to improving CPF (50.50 m, 95% CI 22.23-78.77; *P*<.001), lower limb muscular strength (19.41 kg, 95% CI 11.32-27.49; *P*<.001), and upper limb muscular strength (4.42 kg, 95% CI 0.12-8.73; *P*=0.04) in children with intellectual disability; and (3) the limited research related to this population prevented us from drawing any confirmed conclusions on relationships with obesity.

In relation to the above, we would like to develop a PA intervention to reduce obesity and improve HRPF levels in adolescents with intellectual disability. The proposed study will be a 12-week, school-based PA program with a 2-armed randomized controlled trial (RCT) design. The primary outcomes will be obesity- and HRPF-related outcomes. Blood pressure will be evaluated as a secondary outcome, as some common noncommunicable diseases, such as hypertension and diabetes, that occur in adulthood have become more prevalent in children, mainly due to unhealthy lifestyles and obesity [15]. The objectives of the proposed study are to evaluate the effectiveness of the intervention on obesity- and HRPF-related outcomes (primary outcomes) and blood pressure (secondary outcome) and to evaluate sustainable effects of the intervention on all outcomes, with a comparison with a control group (CG) and adjusted for confounding factors (eg, sociodemographic and lifestyle factors).

## Methods

### Study Design

**Figure 1:**
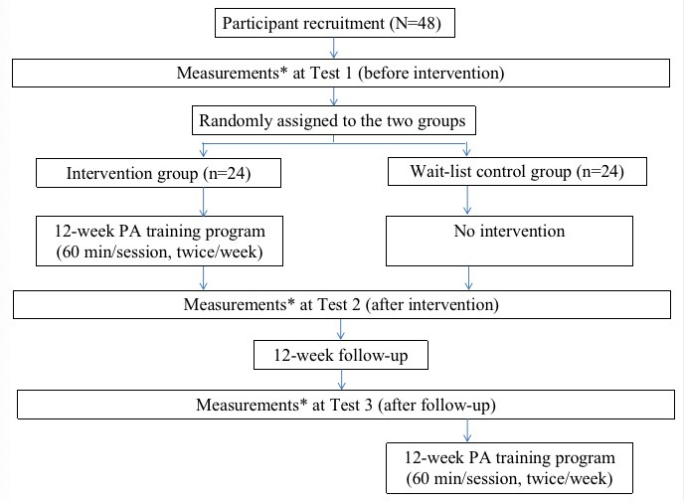
Flow diagram of the study. *Measurements at Tests 1, 2, and 3 will include obesity- and fitness-related outcomes (primary outcomes), blood pressure (secondary outcome), and confounding variables (including demographic information, subjective physical activity [PA] levels, screen time, sleep duration, and eating habits).

The proposed study will be a 12-week, school-based RCT (Figure 1) that will take place in the Chinese mainland. The participants will be recruited from special schools for students with mild intellectual disability and then randomly allocated to either the intervention group (IG) or the wait-list CG. During the 12 consecutive weeks of the study, the participants in the IG will receive a fun game–based moderate-to-vigorous PA (MVPA) training program (2 sessions/week, 60 minutes/session, for a total of 24 sessions). The intensity of the activities will increase in a progressive manner. Those in the CG will receive no intervention. All of the participants (in both groups) will be asked to maintain their regular activities during the intervention period. To observe and evaluate the sustaining effects of the intervention, follow-up testing will be scheduled for all of the participants 12 weeks after the intervention ends. The follow-up period of 12 weeks has been chosen to fit the regular school calendar of the local special schools. The study outcomes will include primary outcomes (obesity- and fitness-related outcomes) and a secondary outcome (blood pressure). In addition, control variables including sociodemographic factors, subjective PA levels, screen time, eating habits, and sleep duration will be collected using a questionnaire. All of the measurements will be taken at 3 time points: Test 1 (T1, before the intervention), Test 2 (T2, immediately following the intervention), and Test 3 (T3, after the 12-week follow-up period). After the follow-up tests, the same PA training program will be provided to the participants in the CG.

### Study Population

#### Target Population and Selection Criteria

The target population of the study will be overweight and obese students aged 12 to 18 years in special schools for those with mild intellectual disability. Overweight and obesity will be defined according to commonly used age- and gender-specific BMI cutoff points (Multimedia Appendix 1) [16]. Participants will be selected according to the following inclusion criteria: (1) children with mild intellectual disability; (2) aged 12 to 18 years; (3) overweight or obese; and (4) at least one family member who is able to attend the program with the participant. The exclusion criteria will be children (1) with a physical disability; (2) with a medical predisposition toward obesity (such as genetic syndrome) that could interfere with the results of the study; (3) with contraindications for PA (eg, severe heart disease); or (4) who participated in other obesity- or fitness-related programs in the past 6 months. This research proposal has been reviewed and approved by the Research Ethics Committee of Hong Kong Baptist University.

#### Sample Size Estimation

The sample size will be calculated using G*Power software (version 3.1.9.4). Previous research has shown that a medium effect size of 0.21 is appropriate [17]. Setting a significance level of *P*<.05, a power level of 80%, and a constant correlation of 0.5, and assuming a 20% dropout rate, a total sample size of 48 participants (24 participants in each group) will be needed.

#### Participant Recruitment

Invitation letters will be sent to special schools in China. If a school agrees to participate, the physical education (PE) teachers at that school will be contacted to help with the initial recruitment of participants by sending a screening questionnaire to their students to determine if they meet the eligibility criteria for study participation. A written informed consent form will be attached to the screening questionnaire to obtain parental consent in advance. Next, the heights and weights of all eligible participants will be objectively measured at their home school. A BMI will be calculated for each student, and students of normal weight will be removed from the list of potential participants. The recruitment will continue until the estimated sample size (n=48) is reached.

#### Randomization and Blindness

We will randomly and equally assign the 48 participants to the IG and CG using block randomization [18]. The random allocation sequence will be computer-generated by a blinded statistician outside of the research term. Masking of participants will be achieved by the wait-list design, where participants assigned to the CG will receive the intervention after the study and therefore will not know that they are in the CG. Furthermore, outcome assessors and statistical analysts will also be masked to the group allocation of the participants.

#### Strategies to Increase Participation and Decrease Dropouts

Previous intervention studies have indicated that parents might refuse to participate if their children do not receive the interventions and thus do not reap the potential benefits of the interventions. To address this issue, we will adopt the wait-list CG design to ensure that all participants will have an equal chance to undertake the PA training program. The PA training program will be free to the participants, and a 100 Chinese Yuan (US $15.45) coupon will be rewarded to the parent of each participant, which will help to increase participation. In addition, the potential health benefits of the project, such as helping participants decrease body weight and improve HRPF levels, will be clearly described to the participants and their parents to increase their interest in participating.

To reduce dropout rates, rewards (eg, stickers) will be given to participants who complete each intervention session. Stationery (eg, pens, erasers) will be given as a reward to participants who achieve an attendance rate of 85% or higher. In addition, only participants who complete the entire study will receive the 100 Chinese Yuan reward, which will also help to reduce withdrawals.

Establishing effective communication channels with parents will increase their confidence in the project. The participants’ health status (eg, weight change) and session performance will be reported to their parents by messages upon request. In addition, the participating schools will serve as a communication bridge between the research team and participants/parents. Maintaining strong communication with the schools’ PE teachers and nurses will also help the research team to understand the health needs of the participants/parents, which may help to decrease dropout rates.

### Description of Intervention

#### Overview of the Program

**Figure 2:**
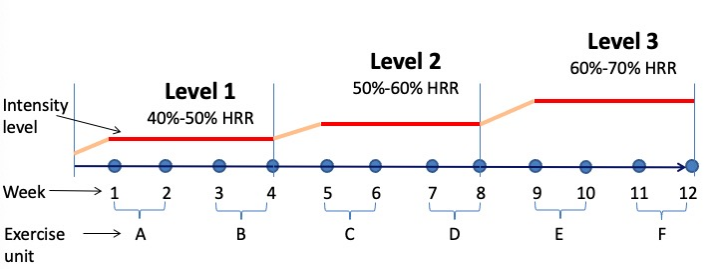
Program overview. HRR: heart rate reserve.

The 12-week PA training program will be delivered at a frequency of 2 60-minute sessions per week, with the exercise intensity increasing progressively from 40% heart rate reserve (HRR) to 70% HRR. Each session will consist of both fun game–based aerobic training and resistance training. In addition, the 12-week PA program will be equally divided into 3 levels with different target exercise intensities, and each level will last for 4 weeks. As shown in Figure 2, the target exercise intensity at levels 1, 2, and 3 will be 40%-50% HRR, 50%-60% HRR, and 60%-70% HRR, respectively. In addition, at the beginning of each level, an intensity adaptation period lasting 2 exercise sessions will be given to the participants to help them adapt to the target exercise intensity.

#### Rationale for Developing the PA Program

The program was designed specifically for children with intellectual disability based on international PA guidelines, exercise guidelines, and evidence from previous PA interventions. The following paragraphs introduce the rationale of the design, including the choice of intervention period, frequency, intensity, time, and content of the proposed program.

##### Intervention Period: 12 Weeks

A duration of 12 weeks has generally been recognized as necessary for an effective PA training intervention [19]; the results of our systematic review of intervention studies for decreasing body weight and improving HRPF levels among children with intellectual disability support this selection. Moreover, attrition rates are lower in short-term interventions than in long-term ones [20]. School activities are usually scheduled on a semester basis, and the most commonly adopted length of time of most school activities is 12 weeks. After reviewing local school calendars, we decided that a short-term period of 12 weeks was the most feasible and practical for implementing the intervention.

##### Frequency: 2 Sessions/Week

Our systematic review revealed that most studies delivered PA training at frequencies of either 2 sessions/week (n=11 [21-31]) or 3 sessions/week (n=9 [25,32-39]). Elmahgoub et al [25] explicitly compared the 2 frequencies and reported that both significantly improved health outcomes. No significant differences in the effects were found between the 2 intervention arms, except for in lower limb muscular strength, which was better in the 3 sessions/week arm. However, the participants’ motivation tended to decline at the end of the program in the arm with 3 sessions/week [25]. As a result, a frequency of 2 sessions/week for PA programs for children with intellectual disability will be used.

##### Intensity: 40%-70% HRR, Progressively Increased

Current PA guidelines [40] suggest that MVPA (40%-89% HRR) provides health benefits to children. However, a high intensity and excessive training load can result in an increased risk of sports injuries, and children with insufficient PA and poor HRPF levels might have difficulty adopting high exercise intensities in the early training stages [41]. Therefore, a progressively increasing exercise intensity, from moderate intensity to vigorous intensity, will be used to help children with intellectual disability achieve health benefits in a safer and progressive way.

##### Time: 60 Minutes/Session

The current PA guidelines suggest that 60 minutes of MVPA per session may help children to achieve health benefits [39]. In addition, our systematic review also revealed that PA programs delivered in 60-minute sessions were more likely to be effective.

##### Type: Aerobic Exercise Combined With Resistance Exercise

Evidence from our systematic review suggested that aerobic exercise combined with resistance exercise was the most effective method for reducing obesity [22,25,27,36,42,43] and improving HRPF levels [25,27,36,38,42,43]. Considering that a fun training mode may improve children’s motivation for participation [44], the proposed study will present aerobic exercise in the form of fun games.

#### Program Content

##### Detailed Content of Each Exercise Unit

**Figure 3:**
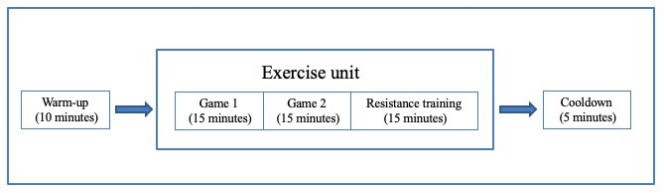
Structure of a training session.

In the program, each training session (see Figure 3) consists of a warm-up (10 minutes), an exercise unit (45 minutes), and a cooldown (5 minutes). Each exercise unit involves 2 different games (15 minutes each) and 3 resistance training exercises (5 minutes each). Each unit will be delivered for 2 continuous weeks. Hence, 6 exercise units with 12 games and 18 resistance training exercises will be prepared.

Content for the 12 games was selected and modified from the Jockey Club Keep-Fit Formula for Children program [45]. Criteria for selection and modification included being (1) safe for children with intellectual disability, (2) easy to learn, (3) fun, (4) able to achieve the intensity requirements, and (5) feasible to perform in special schools. In addition, we have also prepared 6 alternative and replacement games. The resistance training in this program will focus on muscular strength and endurance of children’s upper limbs, abdominals, and lower limbs. Self-weight exercises (eg, push-ups, sit-ups, and jumping jacks) will be adopted for resistance training because they are safer to perform than exercises that use fitness equipment.

To improve the operability, rationality, effectiveness, suitability, and feasibility of the training program, the training contents will be evaluated by two experts before implementation, considering their vast experience in related areas. The experts will consist of a PE teacher in a local special school with more than 5 years of working experience and an active researcher in the research areas of body weight control and/or physical fitness training for at least 5 years. The two experts will be invited to provide their evaluations in terms of feasibility, effectiveness, and suitability of program content. Effectiveness here refers to the content of games and resistance training exercises that will be able to decrease body weight and improve HRPF levels; suitability refers to the training content that will be suitable for the participants to perform without harmful effects; and feasibility refers to the training program that will be able to be practiced in school settings. Two rounds of evaluation will be conducted. The first round will be implemented to gather detailed opinions about each exercise unit from experts using a 5-point Likert scale (ie, 1=very weak, 2=weak, 3=neutral, 4=good, 5=very good); the experts will also be required to provide comments or suggestions for any item with a score of less than 4. After completing the first-round evaluation, modifications will be conducted according to the experts’ opinions and suggestions. If there are any disagreements or queries/confusion about their opinions, a confirmation letter will be sent to them with appropriate explanations. The second-round evaluation refers to the experts’ final evaluation, in which they will be required to give their final assessment of each exercise unit as a pass or fail (scores ≥4 indicate a pass and scores <4 indicate a fail) [46].

Table 1 summarizes the selected and backup games and resistance training exercises. Multimedia Appendices 2 to 7 present the details of exercise units A to F, and Multimedia Appendix 8 presents the six back-up games.

**Table 1:** Details of the exercise units.

Weeks	Exercise unit	Main games and backup games^a^	Resistance training exercises^b^
1-2	A	1. I’m a Tigger 2. Watch me: dribbling and layup (1)^b^ Backup 1: Cross the tunnel	Handgrips (1); jumping jacks (1); sit-ups (1)
3-4	B	1. Jump, jump, throw 2. Tomb robbing Backup 2: Caterpillar	Push-ups (1); squats (1); burpees (1)
5-6	C	1. Cross the river together 2. Obstacle competition (1)^b^ Backup 3: Flip color disks	Handgrips (2); jumping jacks (2); sit-ups (2)
7-8	D	1. Watch me: dribbling and layup (2)^b^ 2. Rapid team (1)^b^ Backup 4: Reaction by color disk	Push-ups (2); squats (2); burpees (2)
9-10	E	1. Step jumping 2. Rapid team (2)^b^ Backup 5: Run and kick	Handgrips (3); jumping jacks (3); sit-ups (3)
11-12	F	1. Obstacle competition (2)^b^ 2. Funny shuttle run Backup 6: Dodgeball	Push-ups (3); squats (3); burpees (3)

^a^See Multimedia Appendix 8 for a detailed description of backup games.

^b^The number in parentheses indicates the level of the game or exercise.

##### Intensity Monitoring and Control

To ensure that the participants’ exercise intensity level reaches the target requirements, we will calculate their target exercise heart rate (HR) using equations 1 to 3 in Textbox 1 [47]. Resting heart rate (HR_rest_) will be measured using an automated device (Omron M6 [HEM-7000-E]; Omron Corporation) at the beginning of each training level. Maximal heart rate (HR_max_) will be estimated using equation 3 in Textbox 1. In each training session, participants’ real-time exercise HR will be calculated two times by tutors by counting the number of beats on their wrists for 10 seconds and then multiplying by six. If the real-time exercise HR is lower than the lower limit of the target range, appropriate modifications (eg, encouraging them to run faster or jump higher) will be adopted to improve their exercise intensity (see Multimedia Appendices 2 to 7). If the exercise HR is higher than the target range’s upper limit, appropriate modifications (eg, encouraging them to run slower or take a rest) will be implemented as well.

**Equations for calculating target exercise heart rate (HR) [47].**

**Equations**

1. Target exercise HR (beats/min) = target % × heart rate reserve (HRR) + resting heart rate (HR_rest_)
2. HRR (beats/min) = maximal heart rate (HR_max_) – HR_rest_
3. HR_max_(beats/min) = 210 – 0.56 × age (in years) – 15.5 × (2 [for children with Down syndrome] or 1 [for children without Down syndrome])

The required exercise intensity for each exercise unit must be estimated. Exercise units with predicted lower intensity will be placed at the beginning of the program (eg, level 1). Over time, the required exercise intensity will increase. The intensity of each exercise unit can be controlled by adjusting the running distance, movement speed, or duration of rest intervals. Specific methods for controlling the intensity of each exercise are given in Multimedia Appendices 2 to 7.

##### Safety Assurance

In each training session, the ratio of tutor to participant will be 1:3. Hence, there will be enough tutors to ensure the participants’ safety. The tutors will pay close attention to participants’ exercise performance to prevent sports injuries. In addition, all of the games are from the game series promoted by the Hong Kong Physical Fitness Association. They are recognized as safer than most self-designed games. As different exercise units may have different characteristics, the safety procedures will vary according to the exercises (see Multimedia Appendices 2 to 7). Furthermore, the equipment used in the program will not have sharp edges. It is better to choose equipment that the participants are familiar with or often used in their PE classes. Moreover, drinking times will be arranged in each training session (eg, participants will drink water every 20 minutes). Finally, in case of emergency, at least one PE teacher from each participant school will be required to attend and assist with each exercise session and on-site data collection.

#### Program Delivery

##### Location

The PA training programs will be conducted in the participants’ schools. A fixed sports area in each participating school, such as a basketball court or the indoor activity venue, will be necessary.

##### Training Time Arrangement

The training program session will be scheduled in a non–PE class time slot during school hours. It will be discussed with each participant school.

##### Ratio of PA Instructors to Participants

To ensure that the participants receive individualized instruction but also have interaction opportunities with their peers [48,49], the proposed study will have a high tutor to participant ratio of at least 1:3 for each training session.

##### PA Instructor Recruitment and Training

The tutors should have some knowledge of PE and have experience leading PE courses for students with special needs. Three training sessions (60 minutes per session) will be provided to all tutors. During the sessions, we will explain the background, objectives, and significance of the study and the characteristics of the participants. Then, we will introduce the interventions and the methods for exercise intensity control and safety for each exercise unit. Finally, all of the study measurements will be introduced.

### Description of Control

Initially, no intervention will be delivered to the participants in the CG during the intervention period. Also, they will not be permitted to join any other related programs. They will be asked to continue their usual school activities. All participants in the CG are expected to receive the same PA program after the completion of this study to ensure that they have the same opportunity to improve their health.

### Measures

Obesity- and fitness-related outcomes will be measured as the primary outcomes. Blood pressure will be a secondary outcome. The participants’ demographic information will be collected at baseline and used as a control in data analysis. Moreover, other potential confounders, such as subjective PA level, screen time, sleep duration, eating habits, and pubertal stage, will also be collected and used as controls in data analysis. All of the measurements, except the demographic data, will be collected at baseline (T1), postintervention (T2), and at the 12-week follow-up (T3). A set of evaluation instruments has been drafted.

#### Primary Outcomes

##### Obesity-Related Outcomes

###### Body Weight

Body weight will be measured with a Tanita body composition analyzer (TBF-410), which is accurate to 0.1 kg. The scale will be cleared before each participant is weighed, and the participants will be asked to look straight ahead and stay still until the digital screen settles before the measurement is recorded. The data reliability can be further increased by using the same scale to measure all of the participants. Body weight will be measured at 3 time points and treated as a continuous variable. Changes in body weight, as an obesity-related outcome, will be calculated and compared between the IG and CG.

###### BMI

BMI scores will be calculated as weight divided by the square of height (ie, kg/m^2^). Measurement of weight is described in the section above. Height will be measured using a height gauge accurate to 0.1 cm (Harpenden Stadiometer, Holtain Ltd). When measuring heights, the participants will be asked to remove their shoes and stand straight, with knees and feet together. BMI will be evaluated at 3 time points and treated as a continuous variable. Changes in BMI, as an obesity-related outcome, will be calculated and compared between the IG and CG. BMI is also a categorical variable, which will be used for overweight and obese classification.

###### Waist Circumference

Waist circumference will be measured midway between the lowest rib margin and the top of the iliac crest at the end of a gentle expiration [50] using a flexible meter ribbon accurate to 0.1 cm. Waist circumference will be measured at 3 time points and treated as a continuous variable. Changes in waist circumference, as an obesity-related outcome, will be calculated and compared between the IG and CG. Waist circumference will also be used to estimate the waist-to-height ratio.

###### Hip Circumference

Hip circumference will be measured at the widest part of the body below the waist [51] using a flexible meter ribbon accurate to 0.1 cm. Hip circumference will be measured at 3 time points and treated as a continuous variable. Changes in hip circumference, as an obesity-related outcome, will be calculated and compared between the IG and CG.

###### Waist-to-Height Ratio

Waist-to-height ratio will be calculated as waist circumference divided by height. It will be evaluated at 3 time points and treated as a continuous variable. Changes in waist-to-height ratio, as an obesity-related outcome, will be calculated and compared between the IG and CG.

###### Body Fat Percentage

Body fat percentage will be estimated with the Tanita body composition analyzer (TBF-410) using foot-to-foot bioelectrical impedance analysis. After entering the participant’s gender, race, height, and age, the participant will be instructed to stand with bare feet on the metal footplates. The data will be displayed automatically. This variable will be estimated at 3 time points and treated as a continuous variable. Changes in body fat percentage, as an obesity-related outcome, will be calculated and compared between the IG and CG.

##### Fitness-Related Outcomes

###### 6-Minute Walk Test

The 6-minute walk test will be used to measure participants’ CPF. The test has been shown to have acceptable validity and reliability for adolescents and young adults with intellectual disability [52]. Testing procedures will follow the study protocol of Chow et al [53]. Participants will be instructed and encouraged to cover the greatest possible distance on a flat surface 25 m in length. They will be instructed to keep a steady pace, whether running or walking. A trained examiner will measure and record the distance covered by each participant. Each participant will be assigned a trained partner (university student helper) based on the participant’s special physical condition and cognitive ability to receive verbal direction and encouragement during the test. The 6-minute walk test will be performed at 3 time points, and the distance covered will be treated as a continuous variable. Changes in distance, as a fitness-related outcome, will be calculated and compared between the IG and CG.

###### Number of Stands in 30-Second Sit-to-Stand Test

A 30-second sit-to-stand test will be used to assess participants’ lower limb strength and endurance. It measures the maximum number of times a participant can rise to a full standing position from a seated position in a 30-second period, without pushing off with their arms. This test is administered using a chair with a seat height of approximately 35.6 cm, without arms. The participants will be instructed to sit with back straight and feet approximately shoulder-width apart and placed on the floor. Arms will be guided to be crossed at the wrists and held against the chest. The number of completed stands will be recorded. This test was first developed for older adults and is highly correlated with strength of the lower limbs [54]. A high correlation of the test score with the strength of the lower limbs (*r*=0.69) has also been found for adolescent children with intellectual disability [33,52]. The number of stands within a 30-second period will be measured at 3 time points and treated as a continuous variable. Changes in the number of stands, as a fitness-related outcome, will be calculated and compared between the IG and CG.

###### Number of Sit-Ups in 1 Minute

A 1-minute sit-up test will be adopted to evaluate participants’ abdominal muscular strength and endurance. The task requires participants to perform as many sit-ups as possible in 1 minute or until the participant experiences muscle fatigue and cannot perform any more. Participants will be instructed to cross their arms on their chests with hands on shoulders, tighten their abdominal muscles, and rise up to touch elbows to thighs. Then, they will return to the start position and repeat [55]. The number of sit-ups completed in 1 minute will be measured at 3 time points and treated as a continuous variable. Changes in the number of sit-ups, as a fitness-related outcome, will be calculated and compared between the IG and CG.

###### Handgrip Strength

The handgrip strength test will be used to assess participants’ hand and forearm muscular strength. During the test, the participant will be instructed to adopt a standing position with arms at his/her side, not touching the body. The participant will be asked to squeeze the instrument with as much force as possible for 10-20 seconds. Right and left hands will be alternated [36]. Handgrip strength will be measured at 3 time points and treated as a continuous variable. Changes in handgrip strength, as a fitness-related outcome, will be calculated and compared between the IG and CG.

###### Sit-and-Reach Distance

For the sit-and-reach test, each participant will begin by removing his/her shoes and sitting down at the test apparatus. One leg will be fully extended with the foot flat against the end of the testing instrument. The other knee will be bent, with the sole of the foot flat on the floor 5 cm to 8 cm to the side of the straight knee. The arms will be extended forward over the measuring scale with hands palms down, one on top of the other. Each participant will be instructed to reach directly forward with both hands along the scale 4 times and to hold the position of the fourth reach for at least 1 second. After measuring one side, the participant will be asked to switch the position of his/her legs and reach again [55]. The distance of the sit and reach will be measured at 3 time points and treated as a continuous variable. Changes in the distance of the sit and reach, as a fitness-related outcome, will be calculated and compared between the IG and CG.

#### Secondary Outcome

##### Blood Pressure

The participants’ systolic and diastolic blood pressure will be measured using an Omron blood pressure monitor (calibrated before using). During the test, the participant will be asked to sit quietly for approximately 5 minutes and then remove outer garments and roll up their shirtsleeves, if necessary, to bare the upper right arm. The measurements will be taken on the right arm. The participant’s arm will be resting on the desk so that the antecubital fossa is at the level of the heart, with the palm relaxed and facing upward. Then, the cuff will be placed on the right arm, with the bottom edge 1 cm to 2 cm above the antecubital fossa. The top edge of the cuff cannot be restricted by clothing. Two measurements will be taken 1 minute apart, and the readings will be recorded. Before and during the measurement, it is necessary for the participant to be calm [56]. Blood pressure will be measured at 3 time points and treated as a continuous variable. Changes in blood pressure will be calculated and compared between the IG and CG.

#### Confounding Variables

##### Subjective Measurements of PA Level, Screen Time, Sleep Duration, Eating Habits, and Pubertal Stage

Subjective data on participants’ PA level, screen time, sleep duration, eating habits, and pubertal stage will be collected using a self-report questionnaire. As the participants may not be able to fully understand questionnaires independently, the participants and their parents will complete the questionnaire together at home. In addition, to increase the reliability of the responses regarding participants’ school activities and snacks, a short version of the self-report questionnaire will be administered to the participant’s schoolteacher. Subjective PA level, screen time, sleep duration, eating habits, and pubertal stage will be collected at 3 time points and treated as confounding variables. They will be used as controls in the data analysis.

The Chinese version of the Global Physical Activity Questionnaire (GPAQ) will be modified to estimate participants’ PA and sedentary behaviors. The modified GPAQ will contain 12 items to ask about the frequencies and duration of moderate and vigorous PA in school, transport, and leisure time situations in a typical week. There will be 2 additional questions about sedentary behaviors in a typical week. Furthermore, we will add questions about screen time (2 items) and sleep duration (2 items) on a typical weekday and on weekends. The average daily time spent performing MVPA will be calculated for the participants and used to divide them into 2 groups (active versus inactive) based on the PA level for children recommended by the World Health Organization (ie, 60 minutes/day). For sedentary behaviors, a cutoff point of 4 hours/day will be adopted to distinguish the 2 groups. The average sleep duration will also be used to divide the participants into 2 groups (sufficient versus insufficient, with a cutoff point of 8 hours/day) [57].

Questions about participants’ eating habits will be adapted from a dietary questionnaire developed by the Central Health Education Unit of Hong Kong for school-aged children [58]. The questionnaire will feature 9 items to evaluate the frequency of food consumption, including the consumption of fruits, vegetables, meats, fish, eggs, beans, grains, fried foods, sweetened drinks, high-sugar foods, high-salt foods, and high-fat foods. The questionnaire will also include 4 items to measure eating habits at breakfast and dinner. In addition, we have adopted questions about snacks (3 items) and fast foods (1 item) from a dietary questionnaire for children that was developed by the Pennsylvania Department of Health [59].

Puberty is a dynamic process of development marked by rapid changes in body composition and health outcomes [60]. Adolescents of the same age may differ in the rate of their physical growth [61]. Research suggests that Tanner staging from a clinical assessment is the gold standard measurement of puberty status [61]. The Tanner staging system consists of 5 stages, separately designed for boys and girls, which are determined by pubic hair growth in both sexes, breast development in girls, and testicular development in boys [62]. However, this kind of assessment is challenging in school settings. As a result, the self-assessment of puberty status might be more acceptable to the participants [60]. In this study, an illustrated Tanner pubertal questionnaire will be provided to participants to collect the information regarding pubertal stage [63].

### Process Evaluation

Process evaluation will be used to monitor and document the implementation of the intervention [61]. A framework model for designing a process evaluation of RCTs will be adopted. Participants’ retention rate, adherence rate, and compliance to the intervention will be assessed. Moreover, all participants will be asked about their satisfaction with the intervention, perceived effectiveness and usefulness of the intervention, future participation intention, and intention to recommend the intervention to others in a 5-point Likert scale questionnaire [64].

### Quality Control

Manual and operational procedures will be prepared for all of the team members. Three training sessions (60 minutes/session) will be provided. Monthly and ad hoc research meetings with experts (university teachers) in the PA area, student helpers, and PE teachers/nurses in the special schools will be held to ensure the success of the proposed study. To promote a uniformly high performance, we will conduct unannounced quality assurance inspections (2 times/month) in the field. The data will be checked by auditors immediately and entered twice to enhance accuracy. Moreover, on the written informed consent form, we will request that the participants in the IG and their parents do not communicate with any parents and classmates in the school about the intervention to avoid contamination and behavioral spillover. In addition, we will seek the participating schools’ help to remind them and monitor and prevent occurrences of such behavior.

### Data Collection Procedure

Once participants have been recruited, informed consent forms and questionnaires will be sent to the participants. They will be completed by the parents with the assistance of the participants within 1 week and returned to their schoolteachers. To ensure the confidentiality of the personal information, these files will be sealed in an envelope. The parents of the participants who do not send back the forms will be contacted via a phone call or text message. New documents will be prepared and sent to the parents if the documents have been lost or mislaid.

After collecting the required documents, we will arrange a morning session to conduct the obesity- and fitness-related tests and blood pressure measures for the participants. The test procedure is as follows: heart rate test and blood pressure measurement first, then the obesity-related tests, and finally the fitness-related tests. The 6-minute walk will be the last fitness test. If a participant is absent on the test day, another test session will be arranged after consultation with the school.

### Data Analysis

SPSS Statistics (version 23.0; IBM Corp) will be used for the data analysis. Mean (SD) and/or median (IQR) values will be calculated for the continuous variables (eg, BMI). All of the continuous variables at T1 will be tested for between-group differences using an independent sample *t* test. Normality will be checked using Q-Q plots and tested for equal variance, as assessed by Levene’s test. If skewed, the data will be logarithmically transformed before the *t* test is applied. A repeated measures analysis of variance will be used to test for significant differences in the changes in the continuous outcome variables between the 2 groups, where group and time are the 2 factors of interest and will be controlled for significant confounding factors (eg, age). The variables will be selected in a stepwise manner, and only those with *P*<.1 will be kept in the final models. A significance level of .05 will be adopted (2-tailed test). Intention-to-treat analysis will be adopted and performed to avoid bias from withdrawals or protocol deviations.

### Pilot Study

A 2-week pilot study in approximately 10 overweight/obese adolescents with mild intellectual disability will be conducted to test the data collection procedures (outcome measurements) and instruments (questionnaires) and to observe the adaptability of the intervention content. Amendments will be made where necessary. The pilot study will also be used to test the intensity of the 12 selected games. If necessary, the game order will be changed or some games may be replaced with backup games to meet the intensity requirements. The participants in the pilot study will not be involved in the main study.

## Results

**Figure 4:**
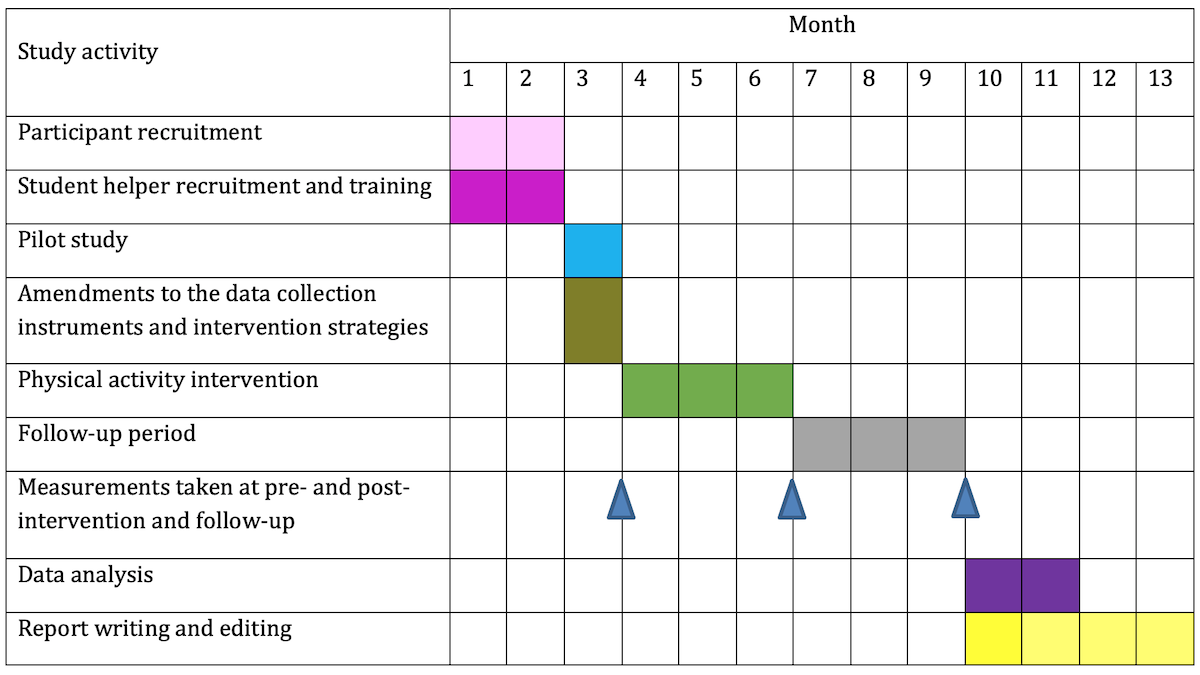
Study timeline.

This study is ongoing. The participants were recruited from October 2020 to November 2020. Total duration of the study is 13 months. Study results are expected at the end of 2021. Figure 4 shows the timeline of the study (by month).

## Discussion

Childhood obesity accompanied with lower levels of HRPF is a major threat to public health both internationally and locally [1]. Children with intellectual disability, especially adolescents, have a higher risk of being overweight/obese and having lower levels of HRPF [9,57]. Therefore, more interventions are needed to help this population attain its optimal health. However, there has been relatively limited research on this population compared with on its TD peers. The proposed PA program will address this knowledge gap by examining the success of the intervention for the target population.

This study has some limitations that need to be acknowledged. First, the PA level is a confounding variable in this study and will be collected in the form of self-reporting because most children with intellectual disability refuse to wear accelerometers, according to our previous experience. Self-reporting methods may result in problems in capturing accurate PA levels. Future studies, therefore, should consider ways to obtain PA levels from children with intellectual disability as accurately as possible. Second, results of this study cannot be applied to children with severe or profound intellectual disability because there may be possible differences associated with different levels of intellectual disability. Third, results of this study may not be applied to children studying in mainstream schools, as school settings between special schools and mainstream schools may be different.

One of the strengths of this study, in our view, is that the PA program was developed based on the results of our systematic review and meta-analysis, which was used to identify effective lifestyle interventions for reducing obesity and/or improving HRPF levels in children with intellectual disability. Other strengths of this study include the use of fun games to improve children’s motivation for participation [44]. To our knowledge, this is the first time that fun games have been adopted to tackle the target health problems in children with intellectual disability. To improve the rationality, suitability, and operability of this program, the contents of the fun games and resistance exercises will be evaluated by experts before implementation, using their vast experience in the related areas. Moreover, the RCT design with sufficient sample size, detailed inclusion and exclusion criteria, and standardized testing may improve the research quality.

If proven effective against obesity and poor HRPF, the intervention design will be made available to the participating special schools so that the intervention can continue to benefit their students. If successful, the program will also be promoted to other local special schools with students with intellectual disability. Furthermore, the study can serve as an example for international researchers, policy makers, and members of the public who are seeking to tackle the problem of obesity and poor HRPF among children with intellectual disability. The aim is to ultimately eliminate the existing health inequities between children with intellectual disability and their TD peers.

## Appendix

Multimedia Appendix 1 International BMI criteria for overweight and obesity by sex for children between 12 and 18 years old.

Multimedia Appendix 2 Details of exercise unit A.

Multimedia Appendix 3 Details of exercise unit B.

Multimedia Appendix 4 Details of exercise unit C.

Multimedia Appendix 5 Details of exercise unit D.

Multimedia Appendix 6 Details of exercise unit E.

Multimedia Appendix 7 Details of exercise unit F.

Multimedia Appendix 8 Details of 6 backup games.

## Abbreviations

CG: control group
CPF: cardiopulmonary fitness
GPAQ: Global Physical Activity Questionnaire
HR: heart rate
HRmax: maximal heart rate
HRPF: health-related physical fitness
HRR: heart rate reserve
HRrest: resting heart rate
IG: intervention group
MVPA: moderate-to-vigorous physical activity
PA: physical activity
PE: physical education
RCT: randomized controlled trial
TD: typically developing
