# Supplementary material for: Effects of a School-Based Physical Activity Intervention for Obesity and Health-Related Physical Fitness in Adolescents With Intellectual Disability: Protocol for a Randomized Controlled Trial
Source: JMIR Res Protoc. 2021 Mar 22;10(3):e25838. doi: 10.2196/25838 (PMC8088867; doi:10.2196/25838)
Supplement: Multimedia Appendix 1 [file resprot_v10i3e25838_app1.docx]

Appendix 1. International BMI criteria for overweight and obesity by sex for children between 12 and 18 years old [16].

| Age (years) | Overweight  Body mass index (25 kg/m^2^) | | Obesity  Body mass index (30 kg/m^2^) | |
| --- | --- | --- | --- | --- |
|  | Males | Females | Males | Females |
| 12 | 21.22 | 21.68 | 26.02 | 26.67 |
| 12.5 | 21.56 | 22.14 | 26.43 | 27.24 |
| 13 | 21.91 | 22.58 | 26.84 | 27.76 |
| 13.5 | 22.27 | 22.98 | 27.25 | 28.20 |
| 14 | 22.62 | 23.34 | 27.63 | 28.57 |
| 14.5 | 22.96 | 23.66 | 27.98 | 28.87 |
| 15 | 23.29 | 23.94 | 28.30 | 29.11 |
| 15.5 | 23.60 | 24.17 | 28.60 | 29.29 |
| 16 | 23.90 | 24.37 | 28.88 | 29.43 |
| 16.5 | 24.19 | 24.53 | 29.14 | 29.56 |
| 17 | 24.46 | 24.70 | 29.41 | 29.69 |
| 17.5 | 24.73 | 24.85 | 29.70 | 29.84 |
| 18 | 25 | 25 | 30 | 30 |
