# Supplementary material for: Effects of a School-Based Physical Activity Intervention for Obesity and Health-Related Physical Fitness in Adolescents With Intellectual Disability: Protocol for a Randomized Controlled Trial
Source: JMIR Res Protoc. 2021 Mar 22;10(3):e25838. doi: 10.2196/25838 (PMC8088867; doi:10.2196/25838)
Supplement: Multimedia Appendix 2 [file resprot_v10i3e25838_app2.docx]

Appendix 2. Details of Unit A.

| Items  *(duration)* | Contents | Rules and descriptions | Intensity control | Safety assurance |
| --- | --- | --- | --- | --- |
| Warm up *(10-minute)* | - Aerobic activities to music | - Warm up (whole body) through a series of simple movements. The participants should try to follow the rhythm of the music. | - Nil | - Nil |
| Game A1  *(15-minute)* | - I am a Tigger | - In groups of two participants, one member jumps across all of the colour discs with both feet. - Then, the jumper picks up a bean bag and clips it between his/her legs. After jumping back, he/she passes it to the second person. - The second person clips the bean bag between her/his legs, jumps across the colour discs with both feet. After putting the bean bag back, she/he jumps back. | - By increasing/decreasing the jumping distance. - By increasing/decreasing group numbers, to decrease/increase the waiting time. | - Pay more attention to knees and ankles in the warm up section. - While the participants are jumping and running, the tutors should follow beside them to prevent falls. - Control the jumping speed from slow to fast to reduce the incidence of sports injuries. |

Appendix 2. Details of Unit A *(continued).*

| Items *(duration)* | Contents | Rules and descriptions | Intensity control | Safety assurance |
| --- | --- | --- | --- | --- |
| Game A2  *(15-minute)* | - Watch me: dribbling and layup (1) | - Participants are divided into several groups. - They dribble a soft volleyball while **walking** around the traffic cones (10 metres distance). - Arriving at the end point, they throw the ball into the basket. - The participant needs to start again if the ball rolls away. | - By increasing/decreasing the walking distance. - By increasing/decreasing group numbers, to decrease/increase the waiting time. | - Make sure to mobilise each body joint during the warm up section. |
| Resistance training  *(15-minute)* | - Handgrip 1   (upper limbs)   - Sit up1   (abdomen)   - Jumping jack 1   (lower limbs) | - Handgrip 1: Squeeze the handgrip ball for 10 seconds for each hand; 3 sets. - Sit up 1: 10 repetitions/set, 3 sets, with 1-minute break between every 2 sets. - Jumping jack 1: 30 seconds/set, 3 sets, with 1-minute break between every 2 sets. | - By increasing/decreasing repetition numbers/duration of each set. - By increasing/decreasing the duration of interval break. | - Tutors should follow beside the participants and protect them from injuries. |
| Cool down  *(5-minute)* | - Stretching | - Stretching of upper limbs, abdomen and lower limbs. | - Nil | - Nil |
