# Supplementary material for: Effects of a School-Based Physical Activity Intervention for Obesity and Health-Related Physical Fitness in Adolescents With Intellectual Disability: Protocol for a Randomized Controlled Trial
Source: JMIR Res Protoc. 2021 Mar 22;10(3):e25838. doi: 10.2196/25838 (PMC8088867; doi:10.2196/25838)
Supplement: Multimedia Appendix 3 [file resprot_v10i3e25838_app3.docx]

Appendix 3. Details of Unit B.

| Items *(duration)* | Contents | Rules and descriptions | Intensity control | Safety assurance |
| --- | --- | --- | --- | --- |
| Warm up *(10-minute)* | - Aerobic activities to music | - Warm up (whole body) through a series of simple movements. The participants should try to follow the rhythm of the music. | - Nil | - Nil |
| Game B1  *(15-minute)* | - Jump, jump, throw | - Six to eight participants in a group. Following the tutor’s instructions (e.g. single-leg jump, two-leg jump or jumping jack), the participants jump across five hula hoops, run to the throw line, pick up a bean bag and throw it into a hula hoop (5 metres distance). Then, they jump across the five hula hoops back to the start point. Then, the next team member repeats the tasks. - The fastest group get three points, the second fastest group gets two points and so on. The total scores of each group are summed plus the number of bean bags that were thrown into the hula hoop. - Three rounds will be recommended. | - By increasing/decreasing the jumping, running and throwing distance. - By increasing/decreasing group numbers, to decrease/increase the waiting time. | - Pay more attention to knees and ankles in the warm up section. - When the participants are jumping and running, the tutors should follow beside them to prevent falls. |

Appendix 3. Details of Unit B *(continued).*

| Items *(duration)* | Contents | Rules and descriptions | Intensity control | Safety assurance |
| --- | --- | --- | --- | --- |
| Game B2  *(15-minute)* | - Tomb robbing | - Divide participants into three or four groups. - Put all of the bean bags in a hula hoop. - The participants need to take the bean bags from the middle hula hoop and put them into their own hula hoop, within one minute. The game is run in the form of a relay race. Each participant can take only one bean bag at a time. - The winner is the group that gets the most bean bags. | - By increasing/decreasing the running distance. | - Make sure to mobilise each body joint in the warm up section. - When the participants are running, the tutors should follow beside them to prevent falls. |
| Resistance training  *(15-minute)* | - Push up 1   (upper limbs)   - Squat 1   (lower limps)   - Burpees 1   (whole body) | - Push up 1: 10-repetitions/set, 3 sets, with 1-minute break between every 2 sets. - Squat 1: 10 repetitions/set, 3 sets, with 1-minute break between every 2 sets. - Burpees 1: 30 seconds/set, 3 sets, with 1-minute break between every 2 sets. | - By increasing/decreasing repetition numbers/duration of each set. - By increasing/decreasing the duration of interval breaks. | - Tutors should follow beside the participants and protect them from sports injuries. |
| Cool down  *(5-minute)* | - Stretching | - Stretching of upper limbs, abdomen and lower limbs. | - Nil | - Nil |
