# Supplementary material for: Effects of a School-Based Physical Activity Intervention for Obesity and Health-Related Physical Fitness in Adolescents With Intellectual Disability: Protocol for a Randomized Controlled Trial
Source: JMIR Res Protoc. 2021 Mar 22;10(3):e25838. doi: 10.2196/25838 (PMC8088867; doi:10.2196/25838)
Supplement: Multimedia Appendix 4 [file resprot_v10i3e25838_app4.docx]

Appendix 4. Details of Unit C.

| Items *(duration)* | Contents | Rules and descriptions | Intensity control | Safety assurance |
| --- | --- | --- | --- | --- |
| Warm up *(10-minute)* | - Aerobic activities to music | - Warm up (whole body) through a series of simple movements. The participants should try to follow the rhythm of the music. | - Nil | - Nil |
| Game C1  *(15-minute)* | - Cross the river together | - Divide participants into several groups, with two members in each group. - Each group will have two hula hoops. The first group member stands in one hula hoop, then puts another hoop in front of himself/herself, and jumps in it. She/he keeps moving forward by jumping into the hula hoops. - After crossing the river, she/he hands the two hula hoops to the other group member. | - By increasing/decreasing the moving distance. - By increasing/decreasing group numbers, to decrease/increase the waiting time. | - Make sure to mobilise each body joint in the warm up section. - When the participants are jumping, the tutors should follow beside them to prevent falls. |

Appendix 4. Details of Unit C *(continued).*

| Items *(duration)* | Contents | Rules and descriptions | Intensity control | Safety assurance |
| --- | --- | --- | --- | --- |
| Game C2  *(15-minute)* | - Obstacle competition (1) | - The participant needs to cross an obstacle (e.g. foam brick) in front of them **foot by foot.** - Pick up a bean bag from the ground. - Throw the selected bean bag into the cylinder beside him/her (two metres distance). - Finally, run to the finish line as fast as possible. | - By increasing/decreasing number of obstacles. - By increasing/decreasing running distance. - By increasing/decreasing group numbers, to decrease/increase the waiting time. | - Make sure to mobilise each body joint in the warm up section. - When the participants are jumping and running, the tutors should follow beside them to prevent falls. |
| Resistance training  *(15-minute)* | - Handgrip 2   (upper limbs)   - Sit up 2   (abdomen)   - Jumping jack 2   (lower limbs) | - Handgrip 2: Squeeze the handgrip ball, 15 seconds for each hand, 3 sets. - Sit up 2.: 15-repetitions/set, 3 sets, with 1-minute break between every 2 sets. - Jumping jack 2: 45 seconds/set, 3 sets, with 1-minute break between every 2 sets. | - By increasing/decreasing repetition numbers/duration of each set. - By increasing/decreasing the duration of interval break. | - Tutors should follow beside the participants and protect them from sports injuries. |
| Cool down  *(5-minute)* | - Stretching | - Stretching of upper limbs, abdomen and lower limbs. | - Nil | - Nil |
