# Supplementary material for: Effects of a School-Based Physical Activity Intervention for Obesity and Health-Related Physical Fitness in Adolescents With Intellectual Disability: Protocol for a Randomized Controlled Trial
Source: JMIR Res Protoc. 2021 Mar 22;10(3):e25838. doi: 10.2196/25838 (PMC8088867; doi:10.2196/25838)
Supplement: Multimedia Appendix 5 [file resprot_v10i3e25838_app5.docx]

Appendix 5. Details of Unit D.

| Items *(duration)* | Contents | Rules and descriptions | Intensity control | Safety assurance |
| --- | --- | --- | --- | --- |
| Warm up *(10-minute)* | - Aerobic activities to music | - Warm up (whole body) through a series of simple movements. The participants should try to follow the rhythm of the music. | - Nil | - Nil |
| Game D1  *(15-minute)* | - Watch me: dribbling and layup (2) | - Divide participants into several groups. - Dribble the soft volleyball and **run** around the traffic cones (10 metres distance). - After arriving at the end point, throw the ball into the basket. - The participant needs to start again if the ball rolls away. | - By increasing/decreasing the running distance. - By increasing/decreasing group numbers, to decrease/increase the waiting time. | - Make sure to mobilise each body joint in the warm up section. - When the participants are running, the tutors should follow beside them to prevent falls. |

Appendix 5. Details of Unit D *(continued).*

| Items *(duration)* | Contents | Rules and descriptions | Intensity control | Safety assurance |
| --- | --- | --- | --- | --- |
| Game D2  *(15-minute)* | - Rapid team（1） | - Divide participants into several groups, with two or three members in each group. Six traffic cones will be arranged for each group. - Group members need to run to pick up one traffic cone, then run back to the start point (shuttle run, 20 meters). - Each member can only pick up one traffic cone at a time. - The winner is the fastest group. | - By increasing/decreasing the running distance. - By increasing/decreasing number of traffic cones. - By increasing/decreasing group numbers, to decrease/increase the waiting time. | - Make sure to mobilise each body joint in the warm up section. - When the participants are running, the tutors should follow beside them to prevent falls. |
| Resistance training  *(15-minute)* | - Push up 2   (upper limbs)   - Squat 2   (lower limps)   - Burpees 2   (whole body) | - Push up 2: 15-repetition/set, 3 sets, with 1-minute break between every 2 sets. - Squat2: 15-repetition/set, 3 sets, with 1-minute break between every 2 sets. - Burpeers2: 45 seconds/set, 3 sets, with 1-minute break between every 2 sets. | - By increasing/decreasing repetition numbers/duration of each set. - By increasing/decreasing the duration of interval break. | - Tutors should follow beside participants and protect them from sport injuries. |
| Cool down  *(5-minute)* | - Stretching | - Stretching of upper limbs, abdomen and lower limbs. | - Nil | - Nil |
