# Supplementary material for: Effects of a School-Based Physical Activity Intervention for Obesity and Health-Related Physical Fitness in Adolescents With Intellectual Disability: Protocol for a Randomized Controlled Trial
Source: JMIR Res Protoc. 2021 Mar 22;10(3):e25838. doi: 10.2196/25838 (PMC8088867; doi:10.2196/25838)
Supplement: Multimedia Appendix 6 [file resprot_v10i3e25838_app6.docx]

Appendix 6. Details of Unit E.

| Items *(duration)* | Contents | Rules and descriptions | Intensity control | Safety assurance |
| --- | --- | --- | --- | --- |
| Warm up *(10-minute)* | - Aerobic activities to music | - Warm up (whole body) through a series of simple movements. The participants should try to follow the rhythm of the music. | - Nil | - Nil |
| GameE1  *(15-minute)* | - Step jumping | - The participants jump (both feet) between two traffic cones. - When completing a jump, the participant needs to touch the side of the traffic cone. - Repeat jumping and touching for 30 seconds. | - By increasing/decreasing the distance between the two traffic cones. - By increasing/decreasing the round duration. - By controlling jumping speed (e.g. completed a jump within 2 seconds). | - Pay more attention to knees and ankles in the warm up section. - When the participants are - jumping, the tutors should follow beside them to prevent falls. |

Appendix 6. Details of Unit E *(continued).*

| Items *(duration)* | | Contents | Rules and descriptions | Intensity control | Safety assurance |
| --- | --- | --- | --- | --- | --- |
| Game E2  *(15-minute)* | - Rapid team（2） | | - Divide participants into several groups, with two or three members in each group. Six traffic comes will be arranged for each group. - Each group member needs to run to pick up one traffic cone, then **finish five squats while lifting the traffic cone with arms stretching forward**. Finally, she/he runs back with the traffic cone to the start point (shuttle run, 20 meters). - Each member can only pick up one traffic cone at a time. - The winner will be the fastest group. | - By increasing/decreasing the running distance. - By increasing/decreasing the number of traffic cones. - By increasing/decreasing group numbers, to decrease/increase the waiting time. | - Make sure to mobilise each body joint in the warm up section. - When the participants are running, the tutors should follow beside them to prevent falls. |
| Resistance training  *(15-minute)* | | - Handgrip 3   (upper limbs)   - Sit up 3   (abdomen)   - Jumping jack 3   (lower limbs) | - Handgrip 3: Squeeze handgrip ball, 15 seconds for each hand, 4 sets. - Sit up 3: 15-repetitions/set, 3 sets, with 30-second break between every 2 sets. - Jumping jack 3: 45 seconds/set, 3 sets, with 30-second break between every 2 sets. | - By increasing/decreasing repetition numbers/duration of each set. - By increasing/decreasing the duration of the interval break. | - The tutors should follow beside the participants and protect them from sports injuries. |
| Cool down  *(5-minute)* | | - Stretching | - Stretching of upper limbs, abdomen and lower limbs. | - Nil | - Nil |
