# Supplementary material for: Effects of a School-Based Physical Activity Intervention for Obesity and Health-Related Physical Fitness in Adolescents With Intellectual Disability: Protocol for a Randomized Controlled Trial
Source: JMIR Res Protoc. 2021 Mar 22;10(3):e25838. doi: 10.2196/25838 (PMC8088867; doi:10.2196/25838)
Supplement: Multimedia Appendix 7 [file resprot_v10i3e25838_app7.docx]

Appendix 7. Details of Unit F.

| Items *(duration)* | Contents | Rules and descriptions | Intensity control | Safety assurance |
| --- | --- | --- | --- | --- |
| Warm up  *(10-minute)* | - Aerobic activities to music | - Warm up (whole body) through a series of simple movements. The participants should try to follow the rhythm of the music. | - Nil | - Nil |
| Game F1  *(15-minute)* | - Obstacle competition (2) | - Each participant needs to **jump** across the obstacle (e.g. foam brick) in front of them **with both feet.** - Then pick up a bean bag from the ground. - Throw the selected bean bag into the cylinder beside him/her (two metres distance). - Finally, run to the finish line as fast as possible. | - By increasing/decreasing the number of obstacles. - By increasing/decreasing the running distance. - By increasing/decreasing group numbers, to decrease/increase the waiting time. | - Make sure to mobilise each body joint in the warm up section. - While the participants are jumping and running, the tutors should follow beside them to prevent falls. |

Appendix 7. Details of Unit F *(continued).*

| Items *(duration)* | Contents | Rules and descriptions | Intensity control | | Safety assurance |
| --- | --- | --- | --- | --- | --- |
| Game F2  *(15-minute)* | - Funny shuttle run | - Three sets of the same items (e.g. colourful bean bags, foam bricks) will be arranged by the tutor and put in front of the participants (15 metres distance). - The tutor picks up several items and lines them in order in the middle of the playground (as an example). - The participants need to run and pick up items that match items in their own sets, then run back and line them up in order. They can only pick up one item at a time. - Two or three participants play at the same time. - The winner will be the fastest participant. | | - By increasing/decreasing the running distance. - By increasing/decreasing the number of items. | - Make sure to mobilise each body joint in the warm up section. - While the participants are running, the tutors should follow beside them to prevent falls. |
| Resistance training  *(15-minute)* | - Push up 3   (upper limbs)   - Squat 3   (lower limps)   - Burpees 3   (whole body) | - Push up 3: 15-repetitions/set, 3 sets, with a 30-second break between every 2 sets. - Squat 3: 20-repetition/set, 3 sets, with a 30-second break between every 2 sets. - Burpees 3: 45 seconds/set, 3 sets, with a 45-second break between every 2 sets. | - By increasing/decreasing the repetition numbers/duration of each set. - By increasing/decreasing the duration of the interval breaks. | | - Tutors should follow beside participants and protect them from sports injuries. |
| Cool down  *(5-minute)* | - Stretching | - Stretching of upper limbs, abdomen and lower limbs. | - Nil | | - Nil |
