# Supplementary material for: Effects of a School-Based Physical Activity Intervention for Obesity and Health-Related Physical Fitness in Adolescents With Intellectual Disability: Protocol for a Randomized Controlled Trial
Source: JMIR Res Protoc. 2021 Mar 22;10(3):e25838. doi: 10.2196/25838 (PMC8088867; doi:10.2196/25838)
Supplement: Multimedia Appendix 8 [file resprot_v10i3e25838_app8.docx]

Appendix 8. Details of six back-up games.

| No | Title | Rules and descriptions |
| --- | --- | --- |
| Back-up 1 | - Cross the tunnel | - Divide participants into several groups, with four to eight members in each group. Have them stand in a line, with their legs apart - The last member needs to roll a basketball between the other participants’ legs, until it reaches the first member. - Then, the first member needs to run to the end of the line, and again roll the basketball forward. - The winner will be the fastest group. |
| Back-up 2 | - Caterpillar | - The participants will be instructed to start in a push-up position. - Then, without moving their upper limbs, they move their feet forward slowly, keeping the knees straight. - They stop when they cannot move their feet forward anymore. Then, they move their upper limbs forward slowly to a push-up position. - The moving distance will be 10 metres. |
| Back-up 3 | - Flip color disk | - Divide participants into two groups, Group A and Group B. Each participant will be given a basketball. The tutor will put some color disks onto the play area. - All of the participants in both groups needs to dribble their basketballs. At the same time, Group A members need to flip the color disks so the back side is up, while Group B members try to keep the front side of the color disk up. - After 5 minutes, count the number of disks facing each direction. - Each round will be limited to no more than 10 participants. |

Appendix 8. Details of six back-up games *(continued).*

| No | Title | Rules and descriptions |
| --- | --- | --- |
| Back-up 4 | - Reaction by color disk | - The tutor puts four color disks in four different directions. - The participants will be instructed to stand in the middle of the color disks. - When the tutor raises a red color disk, the participant needs to run immediately in the right direction and touch the red color disk, then run back to the middle position. |
| Back-up 5 | - Run and kick | - Divide participants into several groups. - Line them up in a starting line. - Each participant needs to run and kick the bean bag, crossing a traffic cone. Then he/she can pass the bean bag to his/her group member, when he/she arrived at the pass line. - The winner will be the fastest group. |
| Back-up 6 | - Dodgeball | - Three participants inside the cycle while seven outside. - The outside participants can move balls into the inside participants. Three balls in total. - The inside participants need to avoid to be hit by the ball. - If be hit, the inside participant should exchange roles with the outside participant. - The balls must be close to the ground. |
